# Imaging the aging brain: study design and baseline findings of the SENIOR cohort

**DOI:** 10.1186/s13195-020-00642-1

**Published:** 2020-06-26

**Authors:** Alexa Haeger, Jean-François Mangin, Alexandre Vignaud, Cyril Poupon, Antoine Grigis, Fawzi Boumezbeur, Vincent Frouin, Jean-Robert Deverre, Marie Sarazin, Lucie Hertz-Pannier, Michel Bottlaender, the SENIOR team, Christine Baron, Valérie Berland, Nathalie Blancho, Séverine Desmidt, Christine Doublé, Chantal Ginisty, Véronique Joly-Testault, Laurence Laurier, Yann Lecomte, Claire Leroy, Christine Manciot, Stephanie Marchand, Gaelle Mediouni, Xavier Millot, Ludivine Monassier, Séverine Roger, Catherine Vuillemard

**Affiliations:** 1 NeuroSpin, Frédéric Joliot Life Sciences Institute, CEA, Paris-Saclay University, Gif-sur-Yvette, France; 2 Department of Neurology, RWTH Aachen University, Aachen, Germany; 3 JARA-BRAIN Institute Molecular Neuroscience and Neuroimaging, Forschungszentrum Jülich GmbH and RWTH Aachen University, Aachen, Germany; 4 Unit of Neurology of Memory and Language, GHU Paris Psychiatry and Neurosciences, Paris University, Paris, France; 5 Paris-Saclay University, CEA, CNRS, INSERM, BioMaps, Service Hospitalier Frédéric Joliot, F-91400 Orsay, France

**Keywords:** Aging, Imaging, Neurodegenerative disease, Biomarker, Prevention, Cognitive decline, Dementia, Alzheimer’s disease, Intra-person across-test variability

## Abstract

**Background:**

Current demographic trends point towards an aging society entailing increasing occurrence and burden of neurodegenerative diseases. In this context, understanding physiological aging and its turning point into neurodegeneration is essential for the development of possible biomarkers and future therapeutics of brain disease.

**Methods:**

The SENIOR study represents a longitudinal, observational study including cognitively healthy elderlies aged between 50 and 70 years old at the time of inclusion, being followed annually over 10 years. Our multimodal protocol includes structural, diffusion, functional, and sodium magnetic resonance imaging (MRI) at 3 T and 7 T, positron emission tomography (PET), blood samples, genetics, audiometry, and neuropsychological and neurological examinations as well as assessment of neuronal risk factors.

**Results:**

One hundred forty-two participants (50% females) were enrolled in the SENIOR cohort with a mean age of 60 (SD 6.3) years at baseline. Baseline results with multiple regression analyses reveal that cerebral white matter lesions can be predicted by cardiovascular and cognitive risk factors and age. Cardiovascular risk factors were strongly associated with juxtacortical and periventricular lesions. Intra-subject across-test variability as a measure of neuropsychological test performance and possible cognitive marker predicts white matter volume and is significantly associated with risk profile. Division of the cohort into subjects with a higher and lower risk profile shows significant differences in intra-subject across-test variability and volumes as well as cortical thickness of brain regions of the temporal lobe. There is no difference between the lower- and higher-risk groups in amyloid load using PET data from a subset of 81 subjects.

**Conclusions:**

We here describe the study protocol and baseline findings of the SENIOR observational study which aim is the establishment of integrated, multiparametric maps of normal aging and the identification of early biomarkers for neurodegeneration. We show that intra-subject across-test variability as a marker of neuropsychological test performance as well as age, gender, and combined risk factors influence neuronal decline as represented by decrease in brain volume, cortical thickness, and increase in white matter lesions. Baseline findings will be used as underlying basis for the further implications of aging and neuronal degeneration as well as examination of brain aging under different aspects of brain pathology versus physiological aging.

## Background

Given the perspective of current demographic trends, neurodegenerative diseases are expected to affect a rising number of people in the future, together with a significantly increased affected lifetime of patients [1]. However, the reasons for the development of neurodegenerative diseases are still of current debate and the detection of early biomarkers predicting brain disease and cognitive impairment is moving more and more into focus of current research [2–5]. In this context, better understanding the physiological processes of aging, their delineation, and turning points into actual brain disease is essential [6]. Aging is a complex process, comprising an interplay of different cell-intrinsic and local as well as environmental factors. Animal studies suggest that influence from the circulatory system can either accelerate or slow brain aging and cognitive function [7, 8]. In humans, autopsy studies on elderly subjects who have not been diagnosed with a neurodegenerative disease reported tau and amyloid deposits [9, 10] and it still stays unclear what causes these deposits and what are their contributions to neurodegeneration. Both the likeliness and extent of cerebral volumetric changes and other structural alterations increase with old age and can be influenced by intrinsic and extrinsic factors such as cardiovascular risk profile [11–13], but their role in the aging process is still the topic of current research [6, 14]. Altogether, studies so far have clearly pointed to a highly increased inter-individual variability of brain aging underlining the importance of exploration and definition of physiological aging in delimitation to cerebral pathology.

In this context, the SENIOR database is the result of a monocentric, observational study aiming at documenting physiological aging in a cohort of elderly volunteers aged between 50 and 70 years old at the time of inclusion who are subjected to annual examinations over a 10-year period. They comprise multimodal magnetic resonance neuroimaging (MRI) sessions at 3 T and ultra-high-field 7 T, positron emission tomography (PET), serology, neuropsychology, anthropometric, audiometry, and neurological examinations, as well as an assessment of cardiovascular risk factors. Investigation of inter-individual physiological aging and identification of predictive biomarkers and risk factors of brain diseases are envisaged at the conclusion of this observational study.

Advanced, exploratory approaches are being evaluated in this study, including high resolution structural and sodium imaging at ultra-high magnetic field. In combination with blood, genetic, and neuropsychological biomarkers, those new neuroimaging methods could help in detecting early metabolic or structural differentiation from healthy aging, opening avenues for the evaluation of early therapeutic interventions at a presymptomatic stage in the future.

We here present the study design and the baseline data of the SENIOR cohort analyzed in regard to cognitive and cardiovascular risk factors as possible predictors of future cognitive decline. In the context of neuropsychological assessment, recent studies have shown that increased intra-subject across-neuropsychological test variability (i.e., the degree to which each person’s performance differs across tests) is related to decreased cognitive function [15–18] and increased risk for incident dementia [19]. In the SENIOR cohort, we evaluate intra-subject across-neuropsychological test variability in the context of alterations of MRI biomarkers and risk factors for neurodegeneration at baseline as a possible cognitive marker.

## Methods

### Study design

**Fig. 1:**
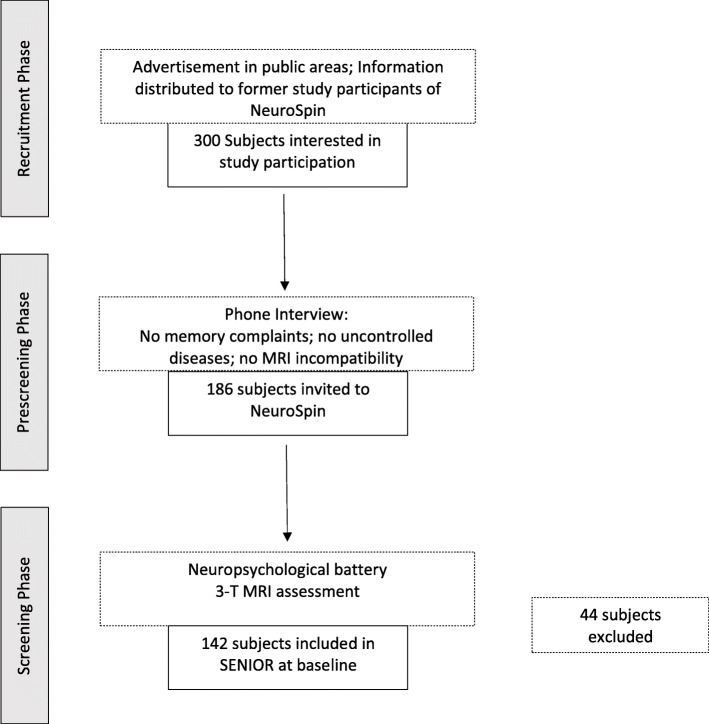
Flowing diagram of inclusion process of the SENIOR cohort

The SENIOR cohort is a study of cognitively healthy volunteers aged between 50 and 70 years at the time of inclusion who agreed on being examined annually over a 10-year period. Three hundred subjects have been initially contacted by the NeuroSpin Center in Saclay, France, between March 2012 and 2017 (inclusion period), indicating interest in participating in the study after public advertisement via flyers and invitations sent to former study participants. Among these volunteers, 186 subjects reporting no memory complaints, uncontrolled chronic diseases, and/or MRI-incompatibility in a pre-screening telephone interview were invited for further neuropsychological assessment and neuroimaging via 3 T MRI for final screening procedure. Among these, a total of 142 subjects were included and performed the complete examination of the baseline visit. Forty-four subjects were excluded for the following reasons: failed to succeed the neuropsychological tests (*n* = 16), detection of structural abnormalities on MRI (*n* = 11), or both (*n* = 3), movement during MRI imaging or artifacts (*n* = 4), not meeting inclusion criteria referring to pre-existing diseases (*n* = 4), or discomfort during the imaging session (*n* = 3) and stopping voluntarily (*n* = 3). An overview of the whole inclusion procedure of the SENIOR cohort is given in Fig. 1. All initially included subjects agreed to be examined once a year for up to 10 years.

### Selection criteria

Detailed inclusion criteria at baseline for the SENIOR cohort were as follows: participants had to be aged between 50 and 70 years at the time of screening, with memory and cognitive assessment in normal range evaluated by a neuropsychologist and without abnormalities such as stroke, tumors, traumatic lesions, hydrocephalus, or artifacts impairing future image analysis in initial 3 T MR imaging (including T_1_, T_2_, T_2_* and FLAIR) according to a neuroradiologist. In detail, scores at the MMSE of > 24 points had to be met for inclusion. Visual and auditory acuity had to be within normal range and informed consent had to be given for study participation. Participants had to be fluent in French language, possess a social security card, and give their written informed consent to study participation.

Exclusion criteria were as follows: contra-indications for high-field MR acquisitions (MR-incompatible implants, intra-ocular or intra-cranial metallic implants, non-removable metallic objects, claustrophobia and significant anxiety, colored tattoos, impossible cooperation, non-removable dental protheses, significant overweight hindering comfortable placing in scanner), any history of intracranial surgery, neurological and/or psychiatric history, chronic disease, alcohol or drug abuse present or up to 2 years before inclusion, non-equilibrated diabetes mellitus, non-controlled arterial hypertension, or cerebrovascular events up to 1 year before inclusion or pregnancy. Participants who develop a vascular pathology as a stroke during the course of the study are taken out from the study. Participants leaving the study for whatever reason are followed whenever agreed via phone interviews about their current life situation and their neurological development via the *Instrumental Activities of Daily Living questionnaire* (IADL). Subjects leaving the study or losing contact during the inclusion period were replaced.

### Study examination

During the screening visit, baseline data were collected, including socio-demographic characteristics and medical history, self-report on current and past alcohol consumption and smoking habits, and current medical treatment with recording of doses and treatment onset. Furthermore, neurological and physical examinations were performed, the latter including anthropometric measurements and measurements of blood pressure and pulse in rest. Subjects underwent a detailed neuropsychological test battery with alternative versions applied each year to avoid learning effects. The test battery included assessment of global cognitive function via *Mini Mental State Examination* (MMSE) and the *Mattis Dementia Rating Scale* [20]. Furthermore, a French adaptation of the *Free and Cued Selective Reminding* test [21] was applied, as well as the *DMS 48* [22], *Rey-Osterrieth Complex Figure* test [23], *Stroop* and *Trail Making Test* [24, 25], French version of the *Frontal Assessment Battery BREF* (FAB) [26], verbal fluency and language assessment via the French oral nominating test *DO80* [27], and apraxia assessment. Subjects further reported their level of physical activity each year via the short version of the *International Physical Activity Questionnaire* (IPAQ) since physical activity can represent a protective factor against cognitive decline [28, 29]. Furthermore, at each annual visit, tone audiometry to detect hearing impairment in the context of cognitive decline [29, 30] is applied by trained nurses in a sound-insulated box using a Harp apparatus (Inventis, Padova, Italy). The right and left ears are tested separately on 9 frequencies (0.25, 0.5, 1, 1.5, 2, 3, 4, 6, and 8 kHz), and the intelligibility of a list of 10 words is tested at 4 intensities (55, 40, 30, and 20 dB).

### Neuroimaging procedure

#### MRI imaging

During the course of the study, subjects were examined once a year at NeuroSpin (Gif-sur-Yvette, France). MRI acquisitions were performed the same day both at 3 T (using a Magnetom Tim TRIO scanner from 2012 to 2015 which was then replaced in the context of a technical upgrade at NeuroSpin with a Magnetom Prisma from 2016 used until now) and a 7 T (using a Magnetom 7 Tesla scanner), all scanners manufactured by Siemens Healthineers (Erlangen, Germany). The improved signal-to-noise ratio (SNR) under ultra-high-magnetic field at 7 T allows to significantly improve the spatial resolution and therefore detection and analyses of small variations in brain substructures, as the hippocampus with its subregions [31] beyond 3 T magnetic field. In addition to the significant improvement in spatial resolution, the additional use of ultra-high-field MRI allows new contrasts induced by magnetic susceptibility effects linked to the presence of iron and gives opportunity to revisit the imaging of non-hydrogen nuclei such as sodium (^23^Na MRI).

Table 1 summarizes the current MRI protocol used at 3 and 7 T. At both magnetic fields, anatomical T_1_- and T_2_-weighted images were acquired (T1 at 3 T: TR = 2300 ms; TE = 2.98 ms; voxel size = 1 mm^3^; T1 at 7 T: TR = 6000 ms; TE = 2.96 ms; voxel size = 0.75 mm^3^). At 3 T, diffusion-weighted images were acquired using the HARDI method [32] (HARDI 1–4 at 3 T: TR = 11,000 ms; TE = 67 ms; voxel size = 2 mm^3^; radial diffusion 1–2: TR = 11,000 ms; TE = 74 ms; voxel size = 2 mm^3^), as well as FLAIR (TR = 9000 ms; TE = 95 ms; voxel size = 1 × 1 × 2.5 mm^3^) and resting-state functional MRI (TR = 2390 ms; TE = 30 ms; voxel size = 3 mm^3^). At 7 T, an additional multi-gradient-echo acquisition was performed to estimate T_2_* maps as well as B_1_+ and B_0_ maps for calibration and correction purposes. Recently, a sodium (^23^Na) ultra-short echo-time acquisition combined with a twisted projection imaging (TPI) k-space sampling scheme applying the variable flip angle method [33, 34] with external sodium phantoms for estimation of brain tissue sodium concentration with a ^1^H/^23^Na volume coil (Rapid Biomedical) was added to the 7-T MRI session in 2019.

**Table 1:** Summary of the current MRI protocols at 3 and 7 T

	Acquisition 3-T MRI		Acquisition 7-T MRI	
Sequence number	Sequence labeling	Sequence duration	Sequence labeling	Sequence duration
1	Localizer	29”	AAH Scout	41”
2	3D T1-weighted	9′14”	T2_loca	32”
3	Resting state fMRI	10′06″	B0 map	54”
4	Diffusion HARDI (total)	15′10″	B1 map	40”
5	Radial Di (total)	9′14″	3D T1 MPRAGE	9′38”
6	B0 map	1′46”	3D T2 STAR	9′48”
7	2D T2 FLAIR	4′05″	SODIUM TPI	31′02”
8	2D T2 GRE	4′06”		
9	2D T2 TSE	2′38”		
10	3D T2 swi	3′31”		

#### PET imaging

In order to measure the cerebral amyloid load of willing participants, a cerebral PET acquisition was performed once at Service Hospitalier Frédéric Joliot (SHFJ, Orsay, France) on a high-resolution tomograph dedicated for neuroimaging (HRRT, Siemens Healthineers).

Amyloid deposition can be one of the early signs of AD and is considered as an early biomarker [26, 35, 36]. However, studies also showed, on the one hand, amyloid depositions in elderlies without memory complaints. On the other hand, amyloid load seems to be indeed associated with structural brain alterations [37]. Therefore, PET imaging has been applied for a better description of the study population and to investigate in a longitudinal way the association between amyloid load and clinical and MRI parameters.

Amyloid-PET dynamic acquisition was performed 40 to 60 min after injection of 341 ± 68 MBq of [^11^C]-PiB. The emission acquisition was preceded by a 6-min brain transmission scan performed using a ^137^Cs point source to correct the emission scan for tissue attenuation. All corrections (attenuation, normalization, random and scatter coincidences) were incorporated in an iterative OSEM reconstruction. The partial volume effect was corrected by directly incorporating resolution modeling (i.e., point spread function modeling) inside the iterative algorithm [38, 39] so that no further post-correction was needed. Ten iterations using 16 subsets were used.

#### Imaging analysis

**Fig. 2:**
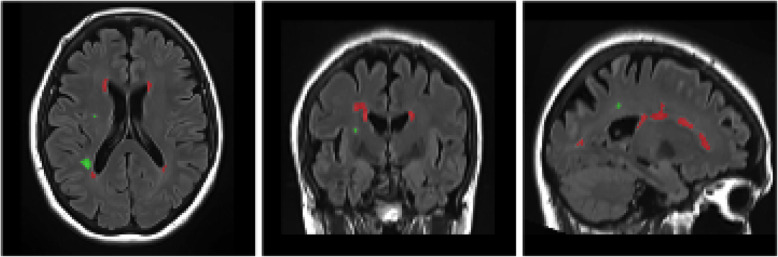
Segmentation of white matter lesions in an exemplary subject of the SENIOR cohort. In red, periventricular lesions, in green, deep white matter lesions are shown

First, T_1_-weighted images acquired at 3 T on the baseline visit were segmented into gray and white matter, CSF, and subcortical structures via the Freesurfer image analysis suite (http://surfer.nmr.mgh.harvard.edu/) [40–45]. Segmentation into cerebral regions of interests and cortical thickness using Killiany/Desikan parcellation atlas was performed. In another step, FLAIR and T_1_-weighted acquisitions were used for the segmentation of white matter lesions with VolBrain Toolbox [46] into whole lesion volume, corresponding to the total volume of all intracerebral lesions, corrected for total intracranial volume (TIV) with differentiation between periventricular, juxtacortical, and deep white matter lesions. Single local lesions were automatically counted in number and summarized into total lesion count. An exemplary subject with segmentation of white matter lesions is shown in Fig. 2.

Amyloid PET imaging analysis was performed as previously described [39, 47]. Briefly, parametric images were created using BrainVisa software (http://brainvisa.info) on averaged images over 40–60 min after injection of [^11^C]-PiB. Standard uptake value ratio (SUVr) parametric images were obtained by dividing each voxel by the corresponding value found in the cerebellar gray matter, used as reference region [39, 47].

The Automated Anatomic Labeling (AAL) Atlas segmented 76 cortical *volume of interest* (VOI) were warped in the T_1_ space of each subject and were intersected with the T_1_ MRI gray matter mask to perform a pseudo-atrophy correction. These VOIs on individual MRI scans were then applied on PET space of each subject after coregistration using a standard mutual information algorithm. We also determined a *global cortical index* (GCI) as defined elsewhere [48]: this VOI includes most of the associative cortical structures. Positivity of amyloid load was defined when GCI was higher than 1.45 [47].

#### Blood samplings

At each annual visit, blood samples are taken which include complete blood count, blood electrolytes, glycemia, glycated hemoglobin, triglycerides, and high- and low-density lipoprotein. Analyses of blood samples are performed by the USPS/LBM (Laboratoire d’Analyses BioMédicales) and the LMM (Laboratoire du métabolisme et du médicament du service de pharmacologie et d’immunoanalyse) of CEA in Saclay. Furthermore, three plasma aliquots are stored at − 80 °C on site for metabolomics and lipidomic analysis that will be performed altogether at the end of the 10 years follow-up by CEA. Ten more plasma aliquots (200 μL each) are also stored accordingly for possible analyses of emerging peripheral biomarker of interests for neurodegenerative diseases.

At the first year visit, a blood sample was withdrawn for genetic analysis and stored at − 20 °C. DNA extraction and *apolipoprotein E* (APOE) genotyping of the three major APOE alleles (ε2, ε3, and ε4) was performed in a subsample of the cohort by AROS Applied Biotechnology (Denmark).

#### Follow-up

An overview of the annual visits is given in Table 2. In summary, subjects receive 3 T and 7 T MR measurements with additional clinical and neuropsychological assessment as well as blood sampling, audiometry, and IPAQ questionnaire every year for a total of 10 years.

**Table 2:**
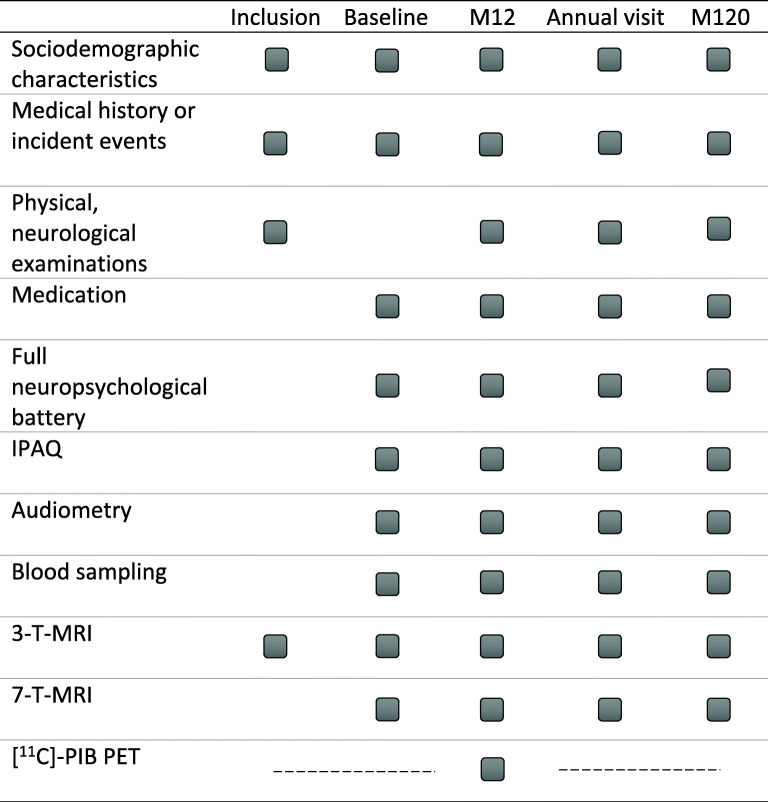
Annual visit and examinations of the SENIOR cohort in months

### Statistical analysis

#### Risk factor and clinical assessment

The following definitions have been used: For education, we considered two categories: obtaining at least French high-school diploma (baccalaureate degree) and/or performing further education (e.g., studies) were considered as high education and otherwise not having obtained this diploma or equivalent considered as lower education. For evaluation of cardiovascular burden, cardiovascular risk factors were counted: diabetes (glycemia > 1.26 g/L, self-reported diabetes or antidiabetic drug intake), hypertension (anti-hypertensive drug intake or self-reported arterial hypertension or increased blood pressure measurements of ≥ 140 mmHg for systolic blood pressure and ≥ 90 mmHg for diastolic blood pressure at a single measurement in rest at visit or occurring systolic or diastolic hypertension in the three consecutive visits), dyslipidemia (plasma cholesterol > 2.5 mmol/L [49], self-reported hypercholesterinemia or use of any lipid-lowering drugs), and active smoking and body mass index (BMI) > 30 at point of inclusion [50]. Each risk factor was evaluated as one point, leading to a maximum of 5 points for all cardiovascular risk factors. Potential depressive symptoms were assessed using the *Geriatric Depression Scale* (GDS) [51]. Neurological examination comprises test for extrapyramidal, pyramidal, cerebellar, frontal syndromes, and visual impairment and was categorized as absent or present; number of copies of the APOE ε4 allele (0, 1, or 2) were evaluated; A cognitive risk factor score was developed by cumulating risk of depression (GDS > 9) [51] and lower education [28, 29, 52, 53], evaluating each risk factor with one point, leading to a maximum of 2 points as cognitive risk factor profile. For whole risk factor profile, cognitive and cardiovascular risk factors were summarized as potentially modifiable risk factors [29].

#### Intra-subject across-neuropsychological test variability

Total absolute scores at the neuropsychological battery were registered and intra-subject across-neuropsychological test variability in performance was calculated based on the method of Holtzer et al. [19] and Gurrera et al. [54] for the neuropsychological assessment at baseline: subtest of different cognitive domains were included, comprising scoring of the MMSE, Mattis Score, Corsi block-tapping (direct and inverse), Digit span (direct and inverse), Grober and Buschke (total recall), DMS (immediate recall), and Rey Figure (immediate and 3-min copy). For assessment of executive functions, Stroop interference and TMT B-A and BREF and for language, phonematic and semantic fluency and the DO 80 score (Table 3) were included. For each subject, the raw scores for each single neuropsychological test score were z-transformed to a *z* score *Z*_*i*_ on the basis of the whole subject sample and used to calculate intra-subject across neuropsychological-test variability *V*_*i*_ of the *i*th subject across all the *K* = 14 neuropsychological subtests:

1 $$ {V}_i=\sqrt{\frac{\sum_{k=1}^K{\left({Z}_{i,k}-{A}_i\right)}^2}{K-1}} $$

**Table 3:** Characteristics of the study sample at baseline. MRI volumetric analyses are corrected for total intracranial volume (TIV) and reported in percent %. Absolute scores of the neuropsychological tests are reported

		Total
*Global information*	Number of subjects	142
	Female, %	50
	Age in years, mean (SD)	60 (6.3)
	Baccalaureate degree or higher education level, %	83.1
	Diabetes or risk, %	5.9
	Hypertension, %	30.3
	Dyslipidemia, %	27.5
	Body mass index, mean (SD)	25.08 (4.4)
	Depression, %	6.3
	At least one APOE ε 4 allele for carried, % (analysis from 109 subjects)	22.9
*Neuropsychology*		
	MMSE score, mean	29.2 (1.0)
*Memory*	Mattis score, mean (SD)	142.3 (2.8)
	Corsi block-tapping, direct and inverse, mean (SD)	5.3 (0.9); 4.7 (0.9)
	Digit span, direct and inverse, mean (SD)	6 (1.1); 4.7 (1.1)
	Grober and Buschke, total recall, mean (SD)	46.6 (1.4)
	Rey Complex Figure Test, immediate copy score, mean (SD)	35.0 (1.4)
	Rey Complex Figure Test, 3-min copy score, mean (SD)	20.5 (7.4)
	DMS48, immediate recall, % (SD)	96.9 (3.7)
	DMS48, delayed recall, % (SD)	97.3 (3.1)
*Executive functions*	TMT A, time in seconds, mean (SD)	31.9 (9.8)
	TMT B, time in seconds, mean (SD)	72.3 (29.8)
	Stroop Interference, time in seconds, mean (SD)	53.8 (11.8)
	BREF, total, mean (SD)	17.1 (1.3)
*Language*	Verbal fluency, mean (SD)	39.0 (9.1)
	Phonematic fluency, mean (SD)	23.9 (6.7)
	DO 80 score, mean (SD)	79.8 (0.7)
	DO 80 paraphasias, mean (SD)	0.1 (0.5)
*Variability*	Intra-person across-test variability	0.89 (0.4)
*MRI Scorings*		
	Fazekas Score^1^, mean (SD)	1.2 (1.1)
	Med. temporal atrophy^1^ R/L, mean (SD)	0.33 (0.53); 0.21 (0.46)
	Enlarged ventricles^1^, % of subjects	13.7
*Volumetric analyses*	Gray matter volume^2^, mean (SD)	47.81 (2.04)
	White matter volume^2^, mean (SD)	35.59 (2.76)
	CSF volume^2^, mean (SD)	16.61 (3.12)
	Hippocampal volume^2^, mean (SD)	0.56 (0.05)
*White matter lesions*	Total lesion count, mean (SD)	10.6 (7.1)
	Total lesion volume^2^, mean (SD)	8.72×10^−4^ (12×10^−5^)
	Periventricular lesion volume^2^, mean (SD)	7.30×10^−4^ (11×10^−5^)
	Juxtacortical lesion volume^2^, mean (SD)	9.01×10^−5^ (1.43×10^−4^)
	Deep white lesion volume^2^, mean (SD)	5.12×10^−5^ (1.19×10^−4^)
*PET (n = 81)*	Index PIB global, mean (SD)	1.28 (0.15)
	Subjects PIB positive (> 1.45), %	8.6%

^1^Visual rating by a neuroradiologist

^2^Corrected for total intracranial volume

*A*_*i*_ is defined as the mean of the *z* scores of one subject across all *K* neuropsychological subtests:

2 $$ {A}_i={\sum}_{k=1}^K\frac{Z_{i,k}}{K} $$

This was performed to evaluate the degree to which each subject’s performance differed across the tasks.

For brain MRI biomarkers, brain regions sensitive to neurodegeneration as the hippocampal volume (by hemisphere), brain parenchymal fraction (gray matter + white matter volumes corrected for total intracranial volume), total white matter lesion (WML) volume, and mean cortical thickness by hemisphere were assessed as well as regional brain volumes, corrected for TIV. Multiple regressions for predictive model assessment was performed with *leave-one-out cross validation* (LOOCV). For model evaluation, bias-corrected *root mean square errors* (RMSE) are reported and consequent ANOVA results of multiple regression analysis.

Further associative analyses between risk factors and structural MRI results included calculation of Spearman rank correlation coefficients *r*_*s*_ and partial correlations *r*. Student’s *t* tests were additionally performed for group comparisons. Statistical results were corrected for multiple comparisons via Holm-Bonferroni correction [55]. Statistical analyses were performed via MATLAB R2018b, R (Version 1.1.463) and SPSS (Version 25).

## Results

### Sample description

**Fig. 3:**
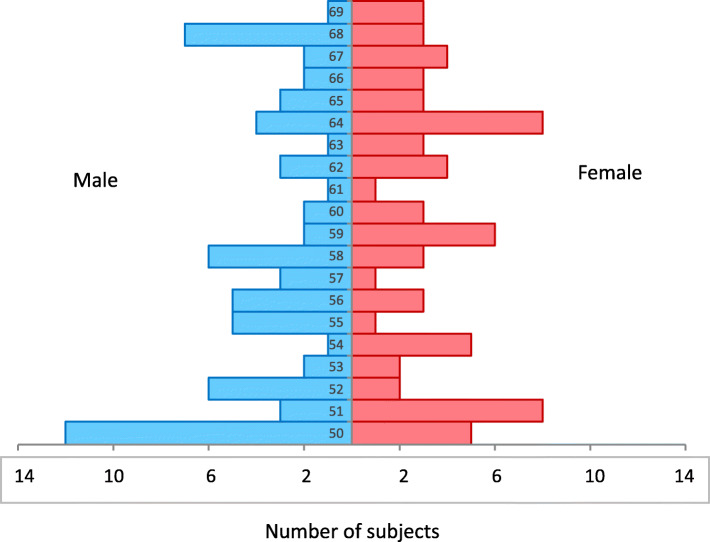
Age pyramid with the total of subjects of the baseline cohort (female in red; male in blue)

Table 3 illustrates the baseline characteristics of the SENIOR cohort: At baseline visit, 142 subjects were included (50% females) with a mean age of 60 (SD 6.3) years; Fig. 3 shows the corresponding age pyramid of all the subjects (female and male) at baseline. One hundred eighteen subjects had an education of at least baccalaureate or higher. Average MMSE at baseline was 29.15 (SD 1.03). GDS was 3.60 (SD 3.80). Concerning cardiovascular risk factors, mean BMI was 25.08 kg/m^2^ (SD 4.43) with 16 subjects with a BMI > 30 (50% females), 43 subjects with arterial hypertension as the most frequently occurring risk factor, 8 self-reported active smokers, 7 with diabetes mellitus and/or glycemia, and 39 with hypercholesterinemia. Cardiovascular risk factors were summarized, leading to 54 participants without known cardiovascular risk factors, 68 with one risk factor, 19 with two or three risk factors, and one with four risk factors. For cognitive risk factors, 111 subjects had no cognitive risk factor, 29 one, and 2 two cognitive risk factors. Twenty-five subjects carried at least one copy of the APOE ε4 allele (only one was APOEε4/ε4). For combined cardiovascular and cognitive risk factors, 46 subjects had no combined risk factors, 56 subjects had one, 33 two, 5 three, one subject had 4, and one 5 risk factors.

For MRI analysis, distributions of mean total gray matter, white matter, and CSF volume in percentage of total intracranial volume of the cohort were 47.81% (SD 2.04), 35.59% (SD 2.76), and 16.61% (SD 3.12). TIV was significantly different in gender (*t (140)* = 6.41; *p* = 2.07×10^−9^), with females demonstrating smaller brain volumes. Increasing age was further associated with a decrease in brain volume, especially showing in total white matter volume decrease (*r* = − 0.41; *p* = 2.3×10^−6^) and cortical gray matter and mean thickness decrease (*r* = − 0.22; *p* = 0.018), and increase in white matter lesions (*r* = 0.39; *p* = 0.001), when controlling for factor gender via partial correlation analyses. Detailed results of the neuropsychological assessment and MRI results at baseline are illustrated in Table 3.

### Association between cardiovascular and cognitive risk factors and white matter lesions at baseline

A multiple regression analysis and LOOCV were performed to predict white matter lesions from cardiovascular risk factors, cognitive risk factors, age, and gender. The best model included all four variables (*RMSE* = 0.0011) (for evaluation of all models, see Supplementary Table S1A). Cardiovascular risk factors and age, cognitive risk factors, but not gender significantly predicted lesion volume (*t* = 2.77, *p* = 0.0064; *t* = 4.44, *p* = 1. 87 × 10^−5^; *t* = 1.97, *p* = 0.051; *t* = 0.074, *p* = 0.94).

When subdividing lesions into detected deep white, juxtacortical, and periventricular lesions, highest association of cardiovascular risk factors was found with juxtacortical (*r*_*s*_ = 0.40, *p* = 4.86×10^−6^) and periventricular lesions (*r* = 0.28, *p* = 0.0025), in comparison to deep white matter lesions which was not significant (*r*_*s*_ = 0.14, *p* = 0.10). Cardiovascular risk factors were further significantly associated with visual Fazekas scoring (*r*_*s*_ = 0.21, *p* = 0.039) and in trend with absolute lesion count *(r*_*s*_ = 0.18, *p* = 0.072) after correction for multiple comparisons.

### Predictors for brain volumes at baseline and association between risk profile and neuropsychological results

**Fig. 4:**
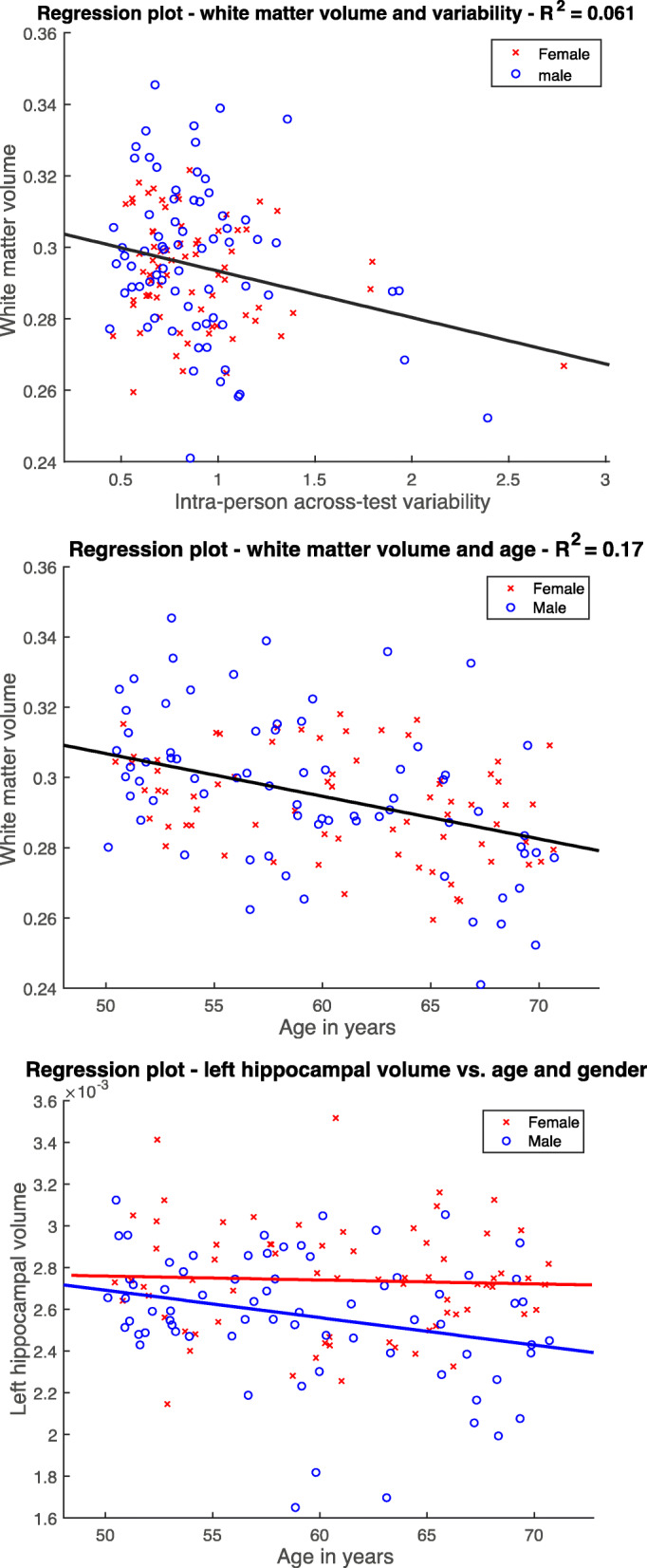
Linear regression plots for white matter and intra-person across-test variability (top), age (middle), and between left hippocampal volume and age and gender for female and male (bottom). In red, female subjects, and in blue, male subjects are illustrated. Volumes are corrected for TIV

We further examined how *V*_*i*_, age, gender, and combined risk factors predicted cerebral volumes at baseline via multiple regression analyses with LOOCV. We hypothesized that these four factors significantly predicted white matter, gray matter, and hippocampal volumes. For white matter volume, best model fit was found for the predictors *V*_*i*_ and age (*RMSE* = 0.0167) (for evaluation of all models, see Supplementary Table S1B), both contributing significantly to white matter volume (*t* = 5.28, *p* = 4.79×10^−7^; *t* = 2.81, *p* = 0.0056) (Fig. 4). For cortical gray matter volume, the best model included age and gender as predictors (*RMSE* = 0.0168), but only age was a significant predictor and gender in trend (*t* = 2.65, *p* = 0.0091; *t* = 1.86, *p* = 0.066). For left hippocampal volume, the best model included age and gender (*RMSE* = 0.000282). Age and gender were significant predictors to left hippocampal volume (*t* = 2.08, *p* = 0.039; *t* = 3.87, *p* = 0.00017) (Fig. 4). For right hippocampal volume, the best model included age and gender as predictor (*RMSE* = 0.000271) but were not significant (*t* = 1.62, *p* = 0.11; *t* = 1.63, *p* = 0.11). Combined risk factors and *V*_*i*_ of our cohort were significantly correlated, when correcting for age and gender (*r* = 0.21, *p* = 0.013).

### Division of cohort group in high and low risk profile and differences in brain volume, cortical thickness, and variability

**Fig. 5:**
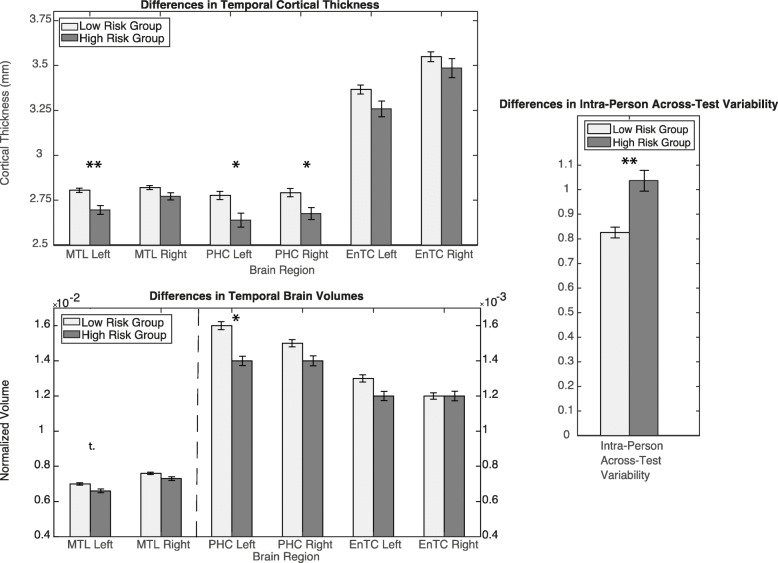
Results of group comparison between high-risk and low-risk group. Effects of factor age were regressed out. MTL (medial temporal lobe), PHC (parahippocampal gyrus), EnTC (entorhinal cortex); *p < 0.05; **p < 0.01; t. (trend *p* < 0.1) after correction for multiple comparisons

We further performed a median split of summarized cardiovascular and cognitive risk factors of the whole cohort, dividing all participants in one group with no or one risk factor and one group with more than one risk factor. Variable age was then regressed out for consequent analyses to compensate for possible age effects on brain volumetry and cortical thickness between both groups. Interestingly, the group with the higher cardiovascular and cognitive risk factor demonstrated a higher *V*_*i*_ as a measure of neuropsychological test performance (*t* (140) = 3.26; *p* = 0.0014). Since we specifically hypothesized an influence on temporal brain structures, we examined cortical thickness and volumetry in temporal regions (medial temporal lobe, parahippocampal region and entorhinal cortex). Cortical thickness displayed a significant difference on left medial temporal lobe and left and right parahippocampal region (*t* (140) = 3.93; *p* = 0.00080 *t* (140) = 3.08; *p* = 0.012; *t* (140) = 2.72; *p* = 0.030) (Fig. 5). For volumetry, left parahippocampal showed significant differences (*t (140)* = 2.67, *p* = 0.043) and left medial lobe in trend (*t* (140) = 1.72; *p* = 0.086). There was no difference in PET-results from GCI comparing the higher and the lower risk profile group (*t* (79) = 0.087; *p* = 0.94).

### Association between PET results and intra-person across-neuropsychological test variability (V_i_)

Since PET acquisition was performed at different time points, we correlated neuropsychological test variability from nearest time point to acquisition with GCI of PET results, which showed no association (*r*_*s*_ = 016, *p* = 0.41). There was further no association between GCI of PET and age (*r*_*s*_ = − 0.15, *p* = 0.45).

## Discussion

We here present our SENIOR study protocol and results at baseline.

SENIOR is a longitudinal, observatory study comprising multimodal assessment via neuroimaging, serology, audiometry, neuropsychology, and clinical examination to evaluate physiological aging processes and the inter-individual variability of physiological versus pathological aging. Improved understanding of these mechanisms during aging will support identification of pathologies in their early, i.e., preclinical stages. The study follows participants yearly for a time period of 10 years, enabling assessment of multiparametric markers of brain pathology and degeneration.

Next to study protocol description and rationale of the protocol, we here present baseline data and discuss these results in the context of aging processes and brain pathology. Multimodal data compiled at the baseline visit were examined in regard to the cardiovascular and potential cognitive risk factors. For neuropsychological analysis, we evaluated the intra-person across-neuropsychological test variability (*V*_*i*_) to overcome ceiling effect in several tests in our healthy volunteers. The results were evaluated in the context of dementia-specific biomarkers of the temporal lobe, white matter lesions, and risk profile. Increased cardiovascular risk factors are associated with increased white matter lesions detected using MRI. We further demonstrate that participants with a higher combined cardiovascular and cognitive risk factor profile show a higher variability *V*_*i*_ and present this neuropsychological parameter as a possible early marker of brain pathology: Being part of a risk group shows altered structural patterns as reduction in volume and cortical thickness in brain regions of the temporal lobe sensitive to neurodegeneration.

Definition of potential risk factors for brain degeneration is one of the key challenges of our present [28]. We demonstrate that in our cohort, risk factor profile is associated with white matter lesions and volumetric and cortical thickness alterations and is reflected in a measure of cognitive test performance. We included both cardiovascular risk factors for neurodegeneration with arterial hypertension, diabetes, hypercholesterinemia, active smoking, and BMI and combined it with a cognitive risk profile of modifiable risk factors [29] consisting of occurrence of depression and a low education profile [28, 56, 57]. Depressive symptoms [58] as well as psychiatric symptoms represent a risk factor for cognitive decline in several aspects [59] which are discussed as causal but also as consequence of cognitive decline [60]. Another risk factor is education [28] which can also be discussed as possible cognitive reserve but is still under current research and its influence as potential risk factor for neurodegeneration is debated in the literature [61, 62]. The APOE ε4 allele status is a non-modifiable risk factor and is associated with an approximately threefold risk of developing AD compared with the more common ε3 allele, whereas the more rare ε2 allele is associated with a decreased risk [52, 63, 64]. Our study population with 22.9% carrying one ε4 allele is therefore higher than the range of ε4 allele distribution reported in literature for France, ranging between 12 and 13% [65, 66]. There are further indications that APOE ε4 modifies the relationship between amyloid load and cognitive function [67, 68]. From 81 subjects of the baseline cohort, who have received PET-imaging so far, 7 are PIB-positive, three of them having at least one copy of the ε4 allele. Division of our group according to their risk profile revealed differences in intra-person across-neuropsychological test variability with higher variability in the high-risk group. Higher variability in test performance has been reported to be associated with progressive cognitive decline and incident dementia [19]. Test variability reflecting inconsistency in performance across tests is likely to decrease years before the possible onset of dementia and due to decline of cognitive functions years before diagnosis of a neurodegenerative disease [18, 69, 70] and could even serve as a potential marker for prodromal Alzheimer’s disease [16]. We show in our cohort that estimation of intra-subject across neuropsychological test-performance is indeed associated with cerebral markers and risk profile and could therefore serve as a predictor of neurodegeneration. One of the further advantages of this method is the avoidance of single test interpretation and dependency with ceiling effects in healthy populations, comprising a large specter of neuropsychological tests for overall performance.

Therefore, observation of development of our cohort on the basis of their cognitive profile assessed via intra-subject across-test variability at baseline will be envisaged to evaluate possible cognitive markers for neurodegeneration. In our cohort, there was no association between PET GCI and age or neuropsychological variability. In literature, positive amyloid PET has often been reported in the absence of cognitive decline and can be present years before cognitive impairment [71]. 8.6% of our cohort undergoing PET was PIB-positive. Prevalence of amyloid-positive scans in cognitively healthy subjects is reported in variable proportions [71], ranging mostly between 10 and 30% [72], variability in reports certainly being influenced by different methodologies, cohort definition, genetics, and environmental conditions. Association between amyloid deposition and cognitive performance is still an aspect of current research. Aβ deposition has a role in neurodegeneration, in association with other factors (as tau-related changes, metabolic alterations, dysconnectivity, white matter changes) [73] and might develop its influence on neuronal cells in interplay, following the amyloid cascade hypothesis [74] with Aβ being present without neuronal injury at the beginning. Therefore, further insight into possible brain alterations with a focus on metabolic alterations, as provided by sodium imaging combined with PET, will deepen understanding of neurodegeneration and physiological aging on a metabolic level before cell degeneration occurs [75, 76].

A limitation to be discussed is the possible selection bias due to participants of our study group who show a high general education background and do not show a very high-risk profile both on cardiovascular and cognitive level. They therefore do not necessarily represent a cross section of the general population. A valuable aspect will be the analyses of the consequent visits to how baseline results lead to inter-individual cognitive outputs and to differences between physiological aging and turning point into cognitive decline. The importance stays in the detection and markers most useful in clinical application which could represent a standard in differentiating physiological aging and its variability differentiating from brain disease [77]. Furthermore, another limitation is the sample size at point of inclusion with 142 subjects who entered the longitudinal protocol, resulting from the selection process, with strict and essential inclusion criteria in respect to study hypotheses. Especially the creation of pathological subgroups during the course of the 10-year study will possibly be restricted, also making associations with other studies about onset of AD and other neurodegenerative diseases essential. However, one of the main assets of this cohort is its detailed characterization via multiple methods, including advanced multimodal imaging methods with PET, 3 T MRI, and even ultra-high-field 7 T MRI, detailed neuropsychological assessment, audiometry, serology, clinical examination, and neuropsychiatric assessment as well as the duration of 10 years, making a priori and a posteriori evaluation during the aging process of this cohort possible.

## Conclusions

We present the protocol and first baseline results of our SENIOR cohort, an observatory study on physiological aging and detection of early biomarkers of brain pathology. We show that risk factors at both cardiovascular and cognitive levels are associated with alterations of both brain anatomy and cognition, demonstrating decrease in volumes and cortical thickness of the temporal lobe and increase in intra-person across-test variability, i.e., at how performance in one subject differed across tests. As an underlying goal of the SENIOR cohort, future development of metabolic and structural brain alterations as well as cognition for differentiating inter-subject differences between physiological aging and brain disease based on different biomarkers will be evaluated.

## Supplementary information

**Additional file 1: Table S1** Model comparison for multiple regression analysis for prediction of White matter lesion volume (A) and White matter, Cortical Gray matter, Left HC and Right HC (B). The selected model with lowest bias-corrected RMSE is represented in bold print.

## Data Availability

The SENIOR study is still an ongoing study with unaccomplished data acquisition. Data of accomplished baseline visits may be made available on request to the corresponding author.

## Abbreviations

AD: Alzheimer’s disease
BMI: Body mass index
GCI: Global Cortical Index
IADL: Instrumental Activities of Daily Living questionnaire
IPAQ: International Physical Activity Questionnaire
LOOCV: Leave-one-out cross validation
MMSE: Mini Mental State Examination
MRI: Magnetic resonance imaging
PET: Positron emission tomography
WML: White matter lesion
RMSE: Root mean square error
TIV: Total intracranial volume
TPI: Twisted projection imaging
