# Supplementary material for: Imaging the aging brain: study design and baseline findings of the SENIOR cohort
Source: Alzheimers Res Ther. 2020 Jun 26;12:77. doi: 10.1186/s13195-020-00642-1 (PMC7320588; doi:10.1186/s13195-020-00642-1)
Supplement: Supplementary file 1 — Additional file 1: Table S1 Model comparison for multiple regression analysis for prediction of White matter lesion volume (A) and White matter, Cortical Gray matter, Left HC and Right HC (B). The selected model with lowest bias-corrected RMSE is represented in bold print. [file 13195_2020_642_MOESM1_ESM.docx]

Table S1) Model comparison for multiple regression analysis for prediction of White matter lesion volume (A) and White matter, Cortical Gray matter, Left HC and Right HC (B). The selected model with lowest bias-corrected RMSE is represented in bold print.

A)

| Models and variables | White matter lesion volume |
| --- | --- |
| (I) Age, Gender, Cardiovasc., cognitive risk factor | **0.00112** |
| (II) Age, Gender, Cardiovasc. risk factor | 0.00113 |
| (III) Age, Gender | 0.00115 |
| (IV) Age | 0.00114 |

| Models and variables | White matter | Cortical Gray matter | Left HC | Right HC |
| --- | --- | --- | --- | --- |
| (I) Age, Gender, Variability, Combined Risk factor | 0.0169 | 0.0170 | 0.000283 | 0.000272 |
| (II) Age, Variability | **0.0167** | 0.0169 | 0.000284 | 0.000273 |
| (III) Age, Gender | 0.0172 | **0.0167** | **0.000282** | **0.000271** |
| (IV) Age | 0.0171 | 0.0168 | 0.000294 | 0.000272 |

B)
